# A Single‐Molecule Liposome Assay for Membrane Permeabilization

**DOI:** 10.1002/anie.202503678

**Published:** 2025-06-05

**Authors:** Krzysztof M. Bąk, Daniel C. Edwards, Dylan George, Bhanu Singh, Ryan Ferguson, Tianxiao Zhao, Kristin Piché, Ariel Louwrier, Scott L. Cockroft, Mathew H. Horrocks

**Affiliations:** ^1^ EaStCHEM School of Chemistry University of Edinburgh Joseph Black Building, David Brewster Rd Edinburgh EH9 3FJ UK; ^2^ IRR Chemistry Hub Institute for Regeneration and Repair University of Edinburgh Edinburgh EH16 4UU UK; ^3^ Stressmarq Biosciences Inc Suite 117‐1537 Hillside Ave Victoria British Columbia V8T 2C1 Canada

**Keywords:** Fluorescence microscopy, Ion channels, Ionophores, Membranes, Single‐molecule studies

## Abstract

Cell membrane disruption is associated with numerous diseases and underlies the activity of various antimicrobial agents. The rapid screening of compounds capable of disrupting or permeabilizing biological membranes is essential to the search for new therapeutic drugs. Here, we present a single‐molecule confocal microscopy assay integrated with fast‐flow microfluidics to study membrane permeabilization in large unilamellar vesicles (LUVs) containing as few as seven dye molecules. This assay eliminates the need for liposome immobilization and achieves detection rates in the range of 1000 vesicles per minute, offering unparalleled sensitivity and detection limits as low as 135 pM, corresponding to just eight permeabilizing molecules per vesicle for active compounds such as ionomycin. It provides a robust platform for investigating membrane‐disrupting agents, including those with antimicrobial properties or implicated in neurodegenerative diseases.

## Graphical abstract

The rapid screening of compounds capable of disrupting or permeabilizing biological membranes is essential to the search for new therapeutics. Here, we present an ultrasensitive single‐molecule confocal microscopy assay combined with fast‐flow microfluidics to study membrane permeabilization in large unilamellar vesicles (LUVs).

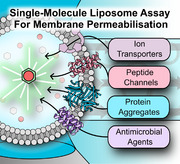

## Introduction

Plasma membrane integrity is vital for cellular health and maintaining ion homeostasis.^[^ ^1^, ^2^ ^]^ Inevitably, membrane disruption is linked to numerous diseases, including Alzheimer's,^[^ ^3^, ^4^, ^5^ ^]^ Parkinson's,^[^ ^6^, ^7^, ^8^ ^]^ and several types of cancer.^[^ ^9^, ^10^, ^11^ ^]^ Membrane permeabilization is also a defense strategy employed by the immune systems to eliminate invading pathogens.^[^ ^1^, ^12^ ^]^ Hence, assessing the ability of compounds to disrupt or permeabilize biological membranes is paramount in the search for new drugs. Small ionophores, which facilitate ion transport across lipid bilayers, induce apoptosis^[^ ^13^, ^14^ ^]^ and exhibit both anticancer^[^ ^15^, ^16^ ^]^ and antimicrobial properties.^[^ ^17^, ^18^ ^]^ Similarly, antimicrobial peptides (AMPs) are promising therapeutic candidates for confronting the challenge of antimicrobial resistance.^[^ ^19^, ^20^, ^21^ ^]^

Membrane permeabilizing agents are typically studied using synthetic lipid vesicles, which serve as convenient models for complex biological membranes. Such vesicles are often employed in fluorescence‐based assays due to their versatility, simplicity, and ease of implementation. The most common approach involves encapsulating self‐quenching fluorescent dyes, such as carboxyfluorescein or calcein, inside large unilamellar vesicles (LUVs, <200 nm in diameter).^[^ ^20^ ^]^ When the membrane of these liposomes is disrupted, the fluorophore leaks out, becomes diluted in the surrounding medium, and its fluorescence recovery can be tracked using a spectrofluorometer. Alternatively, ion‐selective dyes encapsulated within liposomes can detect ion permeabilization by monitoring ion entry into the vesicles.^[^ ^22^, ^23^, ^24^, ^25^ ^]^ Unfortunately, these common spectrofluorometric assays that report on the bulk behavior of vesicle suspensions often require relatively high micromolar concentrations of active compounds (depending on their intrinsic activity) and suffer from reduced sensitivity and selectivity arising from nonspecific dye leakage or mechanically induced membrane disruption.

Microscopy‐based approaches provide exceptional spatiotemporal resolution and allow control over individual liposomes, but typically require giant vesicles (>1 µm) to overcome the diffraction limit of optical methods.^[^ ^26^, ^27^ ^]^ Such vesicles are challenging to obtain and work with under the high ionic strength conditions required for ion transport studies. Single‐molecule fluorescence techniques, such as total internal reflection fluorescence (TIRF) microscopy, enable the investigation of LUV permeabilization at the single‐vesicle level.^[^ ^28^ ^]^ Despite their advantages, current TIRF methods necessitate the immobilization of liposomes on a functionalized surface, which can alter membrane properties, and are limited by low throughput and susceptibility to photobleaching.

**Figure 1:**
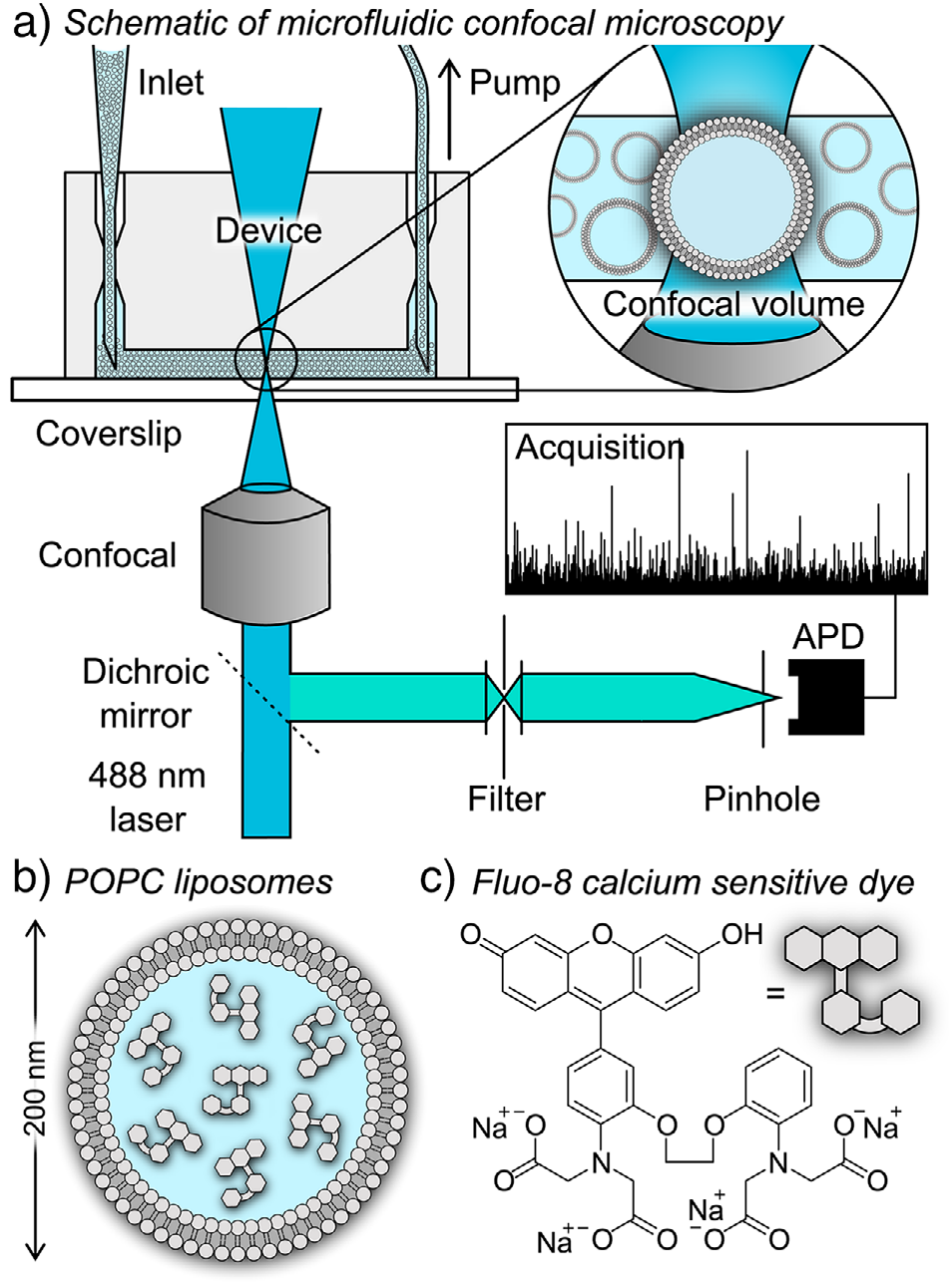
a) Experimental setup, consisting of an inverted confocal fluorescence microscope interfaced with the microfluidic device. Large unilamellar vesicles (LUVs) flow through the confocal volume providing a high detection rate. b) Schematic of 200 nm diameter LUVs composed of POPC lipid and containing on average 7.5 dye molecules of the calcium sensitive dye. c) Chemical structure of Fluo‐8, a turn ON calcium sensitive dye, used in the assay.

**Figure 2:**
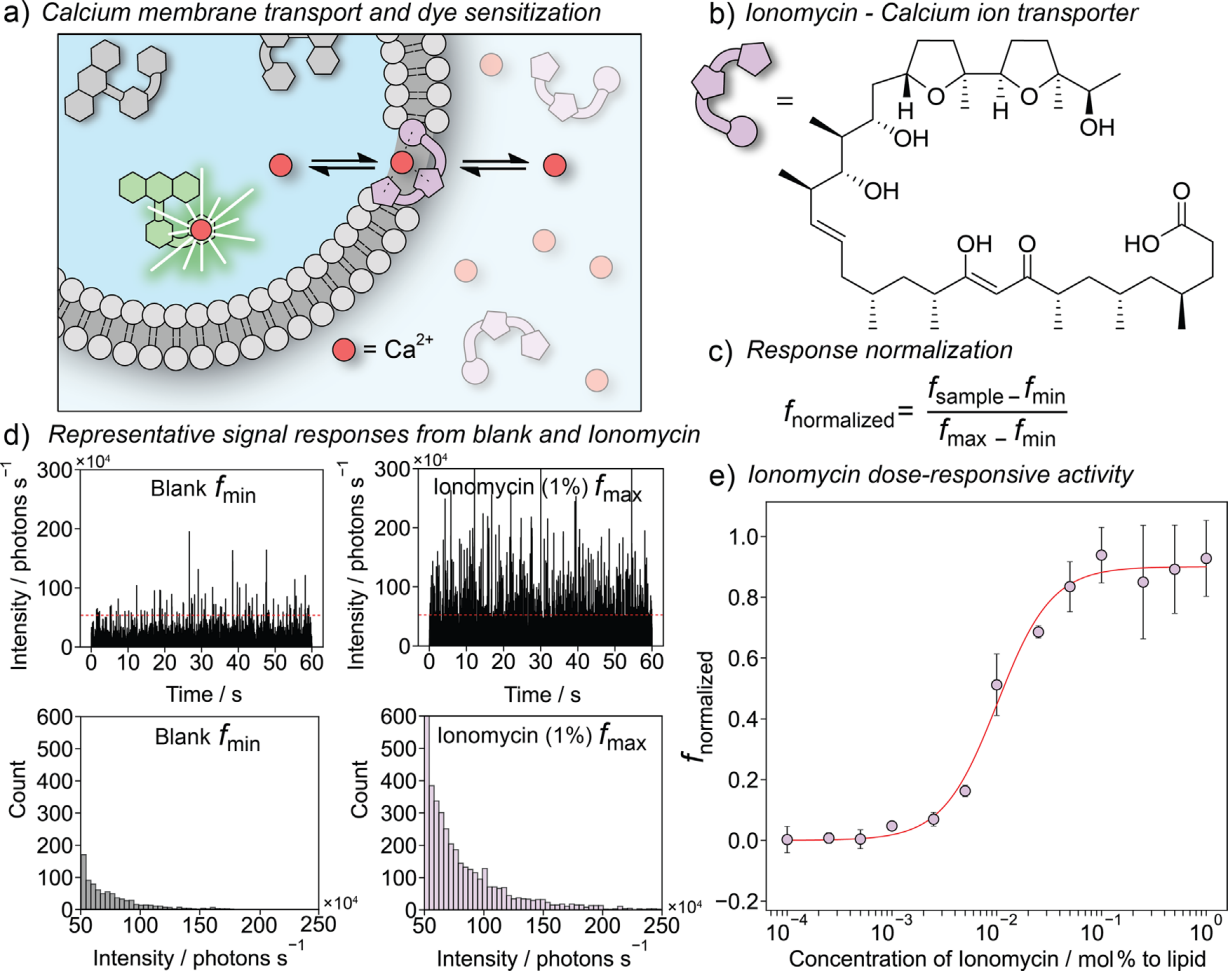
a) Transmembrane permeabilization of Ca^2+^ facilitated by ionomycin. Once within the vesicle, Ca^2+^ sensitizes Fluo‐8 dye that is then detected when in the confocal volume. b) Chemical structure of ionomycin–calcium ion transporter. c) Formula for data normalization used for analysis of fluorescent responses. Normalized fluorescent response, *f*_normalized_, describes activity of the sample; *f*_sample_ is the number of detected events above 50 × 10^4^ photons s^−1^ threshold during 5 min analysis of a sample; *f*_min_ is the number of detected events above 50 × 10^4^ photons s^−1^ threshold during 5 min analysis of a blank sample, which contains CaCl_2_ and no active compound; *f*_max_ is the number of detected events above 50 × 10^4^ photons s^−1^ threshold during 5 min analysis of a sample containing 1 mol% (relative to lipid concentration) of ionomycin. d) Representative 60 s visualization of fluorescence intensity of a solution of large unilamellar vesicles (5 µM), CaCl_2_ (2 mM) containing no ionomycin (left), and 1 mol% (relative to lipid concentration) of ionomycin (right), and their corresponding histograms (below). e) Dose–response curve of ionomycin obtained from three independent experiments. Points show mean ± standard deviation of the sample (*n* = 3). The red curve represents the Hill equation fit.

**Figure 3:**
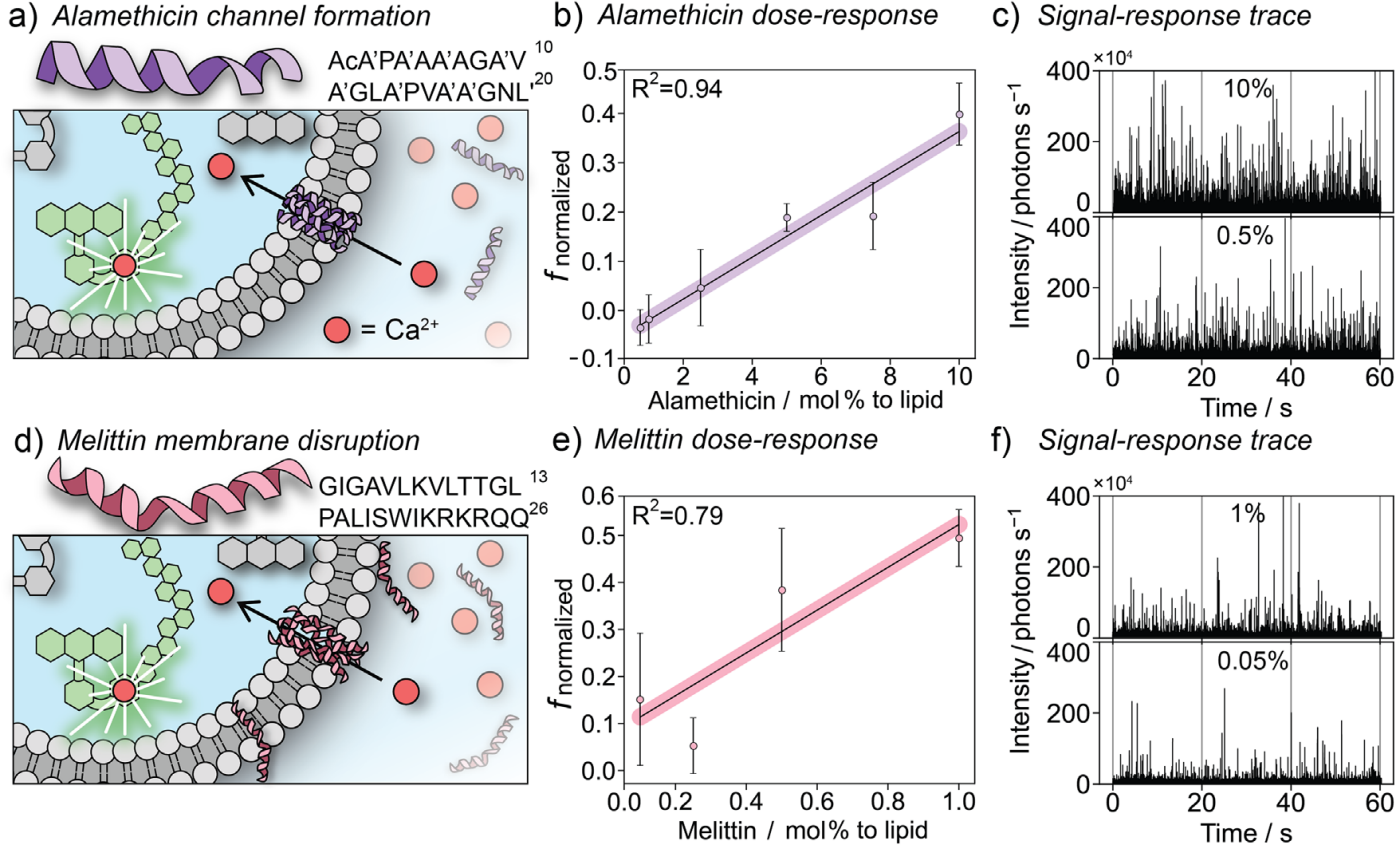
a) Schematic for membrane permeabilization with the barrel‐stave pore‐forming peptaibol alamethicin. Transport of Ca^2+^ sensitizes the Cal‐520 dextran‐conjugated dye. b) Dose–response curve of alamethicin obtained from three independent experiments. Points show mean ± standard deviation of the sample (*n* = 3). c) Representative 60 s visualization of fluorescence intensity for samples containing 10 mol% and 0.5 mol% of alamethicin (relative to lipid concentration). d) Schematic for membrane permeabilization with the membrane‐disrupting peptide melittin. Transport of Ca^2+^ sensitizes the Cal‐520 dextran‐conjugated dye. e) Dose–response curve of melittin obtained from three independent experiments. Points show mean ± standard deviation of the sample (*n* = 3). f) Representative 60 s visualization of fluorescence intensity for samples containing 1 mol% and 0.05 mol% of melittin (relative to lipid concentration).

**Figure 4:**
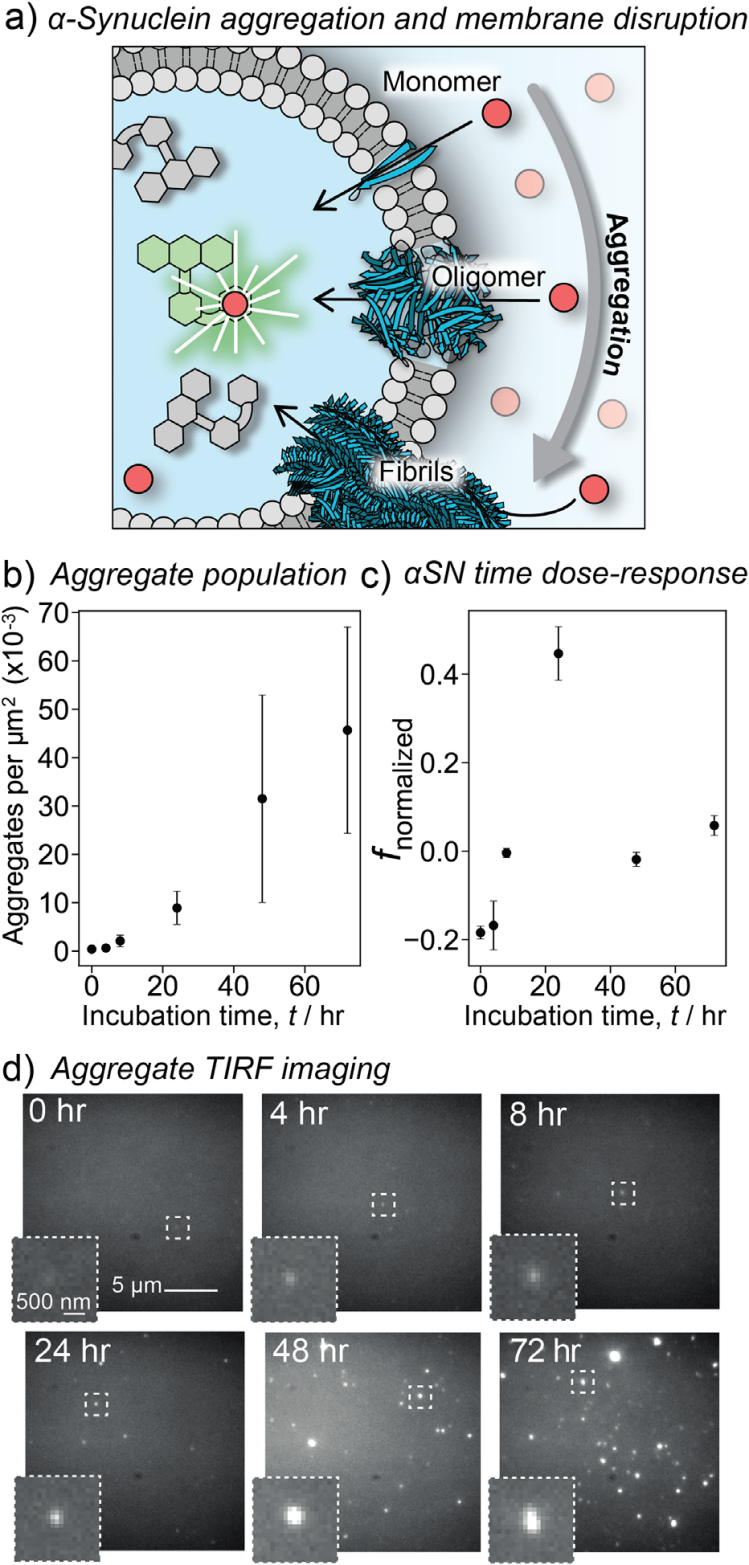
a) Schematic for permeabilization with monomers and aggregates of α‐synuclein. Transport of Ca^2+^ sensitizes Fluo‐8 dye. b) Number of events detected in single aggregate visualization by enhancement (SAVE) imaging of α‐synuclein at different incubation times. c) Membrane permeabilization with aggregates of α‐synuclein at different incubation times. d) SAVE images of α‐synuclein at different incubation times with addition of thioflavin‐T (ThT).

Here, we present an alternative approach by combining single‐molecule confocal microscopy with fast‐flow microfluidics to study the permeabilization of easily obtainable LUVs, eliminating the need for their immobilization (Figure 1).^[^ ^29^ ^]^ By detecting the fluorescence from dye‐loaded vesicles as they transit through a femtoliter‐sized confocal volume, our approach achieves detection rates close to 1000 vesicles per minute—two‐orders of magnitude higher than other single‐molecule approaches.^[^ ^28^, ^30^, ^31^, ^32^ ^]^ The assay enables quantification of concentration‐dependent membrane permeabilization from simple ionophore molecules (Figure 2) to multicomponent protein nanopores. Its broad applicability is further demonstrated by visualizing the effects of antimicrobial peptides (AMPs, Figure 3) and Parkinson's disease‐related oligomers (Figure 4) on lipid membrane integrity. Overall, our approach not only enables the quantification of permeabilization in individual vesicles by very small numbers of molecules but also promises to facilitate high‐throughput drug screening through scale‐up and parallelization.

## Results and Discussion

To investigate membrane permeabilization at the single‐vesicle level, we employed single‐molecule confocal microscopy to track dye‐loaded vesicles as they rapidly pass through a femtoliter‐sized confocal volume (Figure 1a). In our studies, LUVs were composed of 1‐palmitoyl‐2‐oleoyl‐*sn*‐glycero‐3‐phosphocholine (POPC) and filled with the Ca^2+^‐sensitive Fluo‐8 dye (Figure 1c). The LUVs were prepared following a standard procedure involving the hydration of a dry lipid film with HEPES buffer (pH 6.85–7.00) containing the dye, followed by freeze–thaw cycles, and extrusion through track‐etched polycarbonate membranes with 200 nm pores. Nonencapsulated dye molecules were removed by size exclusion chromatography and final lipid concentration of the vesicle suspension was measured by the ^1^H NMR assay using 3‐(trimethylsilyl)propionic‐2,2,3,3‐*d_4_* acid sodium salt (TMSP) as the concentration standard.^[^ ^33^ ^]^ The vesicle sizes were confirmed by dynamic light scattering (DLS) and nanoparticle tracking analysis (NTA) to be in 160–180 nm range (Sections S1.2 and S1.3), corresponding to an average volume of 2.5 × 10^−18^ L (attoliter). LUVs were assembled in the presence of 5 µM dye, hence each vesicle contained, on average, 7.5 dye molecules. The stock vesicle suspension was further diluted with HEPES buffer to obtain a lipid concentration of 5 µM, corresponding to approximately 16 pM of liposomes (Section S1.4).

The diluted vesicle suspension was analyzed using single‐molecule confocal microscopy. Fast‐flow microfluidics directed the vesicle suspension through the confocal volume, which enabled the fast data acquisition rate and limited the effects of photobleaching. Microfluidic channels were fabricated using standard soft‐lithography techniques using polydimethylsiloxane (PDMS) and SU‐8 photoresist on silicon masters, as described previously.^[^ ^29^, ^34^, ^35^ ^]^ The channels (100 µm wide, 25 µm high, and 1 cm long) were plasma‐bonded to glass coverslips to create sealed devices (Figure 1a). The channels were connected to a syringe pump on one side and to a gel‐loading pipette containing ≈100 µL of the LUV suspension analyte at the inlet port. Withdrawing the sample using a syringe pump ensured accurate control of the flow‐velocity and limited the dead volume. The device was then mounted on a confocal microscope equipped with 488 nm laser excitation, which was focused to a diffraction‐limited confocal spot within the channel. The emitted fluorescence was passed through a dichroic mirror, before being focused by a tube lens within the microscope body through a 50 µm pinhole onto an avalanche photodiode (APD) detector, which counts individual photons that are combined into time‐bins corresponding to the expected residence of the LUVs in the probe volume. Our approach achieves detection rates close to 1000 vesicles per minute—two‐orders of magnitude higher than TIRF‐based assays.

As a highly hydrophilic cation, Ca^2+^ cannot freely cross the lipid bilayer. However, the liposome‐entrapped Fluo‐8 dye can be exposed to Ca^2^⁺ in the presence of a lipid‐permeabilizing agent (Figure 2). The fluorescence emission of Fluo‐8 is significantly enhanced upon complexation of Ca^2^⁺; therefore, permeabilized vesicles should produce larger bursts of fluorescence as they pass through the confocal volume. The fluorescence intensity showed occasional increases during 5‐min recordings of the vesicle suspension in HEPES buffer, which were attributed to liposome‐entrapped Fluo‐8 in its uncomplexed form (Figure S8). The addition of calcium chloride solution (2 mM final concentration) to the LUV suspension resulted in a slight increase in the frequency of fluorescent bursts (Figure 2d). This increase may be due to the exposure of residual unencapsulated dye to calcium ions or the penetration of cations into defective, partially leaky liposomes. To induce calcium transport into the liposomes, we used ionomycin, which is a well‐established calcium ionophore. Adding ionomycin (1 mol% relative to POPC) to the calcium chloride‐containing LUV suspension led to a significant increase in the intensity of fluorescence bursts following a 5‐min incubation (Figure 2d). To determine the frequency of events, we counted the number of bursts exceeding a threshold of 50 photons per bin (equal to 50 × 10^4^ photons s^−1^) over the 5‐min period. This threshold value is ∼150 times higher than the average fluorescence intensity of the LUV suspension lacking calcium chloride and ionomycin, which ensured that the signal was well‐distinguished from background noise. In a representative example, the number of events in the 5‐min observation time increased from ∼400, for the LUV suspension, to ∼570 after the addition of calcium chloride and then to ∼3200 after the further addition of ionomycin.

To confirm that the observed signal was a result of Ca^2^⁺ transport rather than dye leakage, Triton X‐100 surfactant was added to disrupt the vesicles and release the encapsulated dye. After liposome rupture, only sparse and weak fluorescence bursts were observed and no event counts above the threshold were detected (Figure S13). Upon release, the original 5 µM Fluo‐8 within the vesicles is diluted ∼100 000‐fold, which significantly reduces the probability of dye molecules entering the confocal volume.

The Ca^2+^ transport properties of ionomycin were further analyzed by conducting dose–response studies in three independent experiments. The number of events in the blank sample containing CaCl_2_ depends on the batch of LUVs. To compare data between different batches, a normalization procedure was applied (Figure 2c) to remove dependence on the absolute number of events. The normalized values represent the change in the number of events between the sample and the blank relative to the change in the number of events between fully permeabilized liposomes and the blank. Full permeabilization of liposomes was achieved by the addition of 1 mol% of ionomycin relative to lipid. The EC_50_ value of ionomycin, which represents the concentration required to elicit a response halfway between the baseline and maximum, was determined to be 0.010 ± 0.001 mol% (Figure 2e). It is worth noting that the EC_50_ value depends not only on the transport activity of the compound but also on its deliverability, which may be reduced at low concentration of LUVs. Nevertheless, the assay detected activity at concentrations as low as 0.0027 mol% or 135 pM of ionomcycin, corresponding to an average of ∼8 molecules per vesicle (Section S2.5). Given the small solution volume used for measurements (100 µL), only 15 fmol of material was needed to record a data point at the limit of detection. The high sensitivity and low detection limits of the developed assay make it ideally suited for studying the membrane‐permeabilizing properties of valuable substances, such as peptides, proteins, natural products, and candidate therapeutics.

Alamethicin and melittin were selected as model antimicrobial peptides to test our assay. Alamethicin monomers can reversibly insert into lipid bilayers and oligomerize to form barrel‐stave pores that allow the translocation of water and ions (Figure 3a).^[^ ^36^, ^37^ ^]^ The addition of 10 mol% alamethicin to the LUVs containing Fluo‐8 led to the disappearance of the detected events, which was attributed to dye leakage. To improve dye retention, Fluo‐8 was replaced by another calcium‐sensitive probe, Cal‐520 conjugated to dextran (*M*_w_ = 10 000 Da). The Stokes’ radius of the selected dextran (∼23 Å) exceeds the size of pores formed by alamethicin (5–11 Å in diameter). This modification enabled us to measure the concentration‐dependent activity of alamethicin (Figure 3c). It was observed only at concentrations >1 mol% and increased steadily, reaching *f*_normalized_ ≈ 0.45 at 10 mol% (Figure 3b).

Experimental and simulations data suggest that melittin forms transient 10–60 Å pores in membranes, with the assembly mechanism depending upon the experimental conditions (Figure 3d).^[^ ^38^ ^]^ Similar to alamethicin, the leakage of Fluo‐8 was observed after the addition of melittin to the LUV suspension. However, Cal‐520 dextran‐enabled observation of calcium transport at >0.1 mol% melittin (Figure 3f). The higher variance in the activity of melittin compared to alamethicin may reflect the previously reported mechanistic variation observed under different experimental conditions (Figure 3e).^[^ ^38^, ^39^, ^40^, ^41^ ^]^

Having successfully assayed the membrane‐perturbing activities of alamethicin and melittin peptides, we sought to broaden the scope by examining proteins associated with neurodegenerative diseases. α‐Synuclein is a neuronal protein that regulates synaptic vesicle trafficking and subsequent neurotransmitter release.^[^ ^42^ ^]^ Oligomeric species arising during the aggregation of α‐synuclein are implicated as a major source of toxicity in Parkinson's disease.^[^ ^43^ ^]^ We used the confocal assay with Fluo‐8‐loaded LUVs to analyze permeabilization properties of α‐synuclein at different stages of aggregation (Figure 4). Monomeric α‐synuclein was incubated under conditions favoring its aggregation for up to 72 h,^[^ ^34^ ^]^ and samples were removed at a series of time‐points for analysis with our permeabilization assay (Section S1.7). Addition of 1 mol% α‐synuclein incubated for 0 and 4 h (50 pM, final concentration) to LUVs resulted in a drop in the frequency of events compared to the background sample (*f*_normalized_ ≈ −0.2, Figure 4c). This suggested partial disruption of the liposomes by monomeric α‐syn. Increase of activity was observed for the sample collected after 8 h, which further peaked at *f*_normalized_ ≈ 0.45 for aliquots collected after 24 h of incubated aggregation (Figure 4c). However, the permeabilization activity dropped back to baseline levels (*f*_normalized_ = 0–0.1) for the aliquots collected after 48 h and 72 h.

To determine the nature of the species causing Ca^2^⁺ influx, we characterized the collected oligomer samples using single‐aggregate visualization by enhancement (SAVE) imaging.^[^ ^44^ ^]^ This method leverages the binding of the benzothiazole dye thioflavin‐T (ThT) to amyloid structures, resulting in a dramatic fluorescence intensity increase, making it a highly sensitive and efficient reporter of extended β‐sheet structures. To detect individual protein aggregates, we employed TIRF microscopy, which enabled counting of individual ThT‐bound aggregates (Figure 4d and Section S1.8). During the first 8 h of the aggregation process, the protein sample primarily consisted of monomeric species, with only a few low‐intensity fluorescent puncta observed. By 24 h, the surface density of aggregates increased to 0.01 aggregates per µm^2^ (Figure 2b). Further incubation for 48 and 72 h resulted in a notable increase in both the number and fluorescent intensity of puncta, indicating the formation of larger oligomers and fibrils. These findings suggest that earlier aggregates of α‐synuclein are responsible for the permeabilization of POPC lipid membranes to Ca^2^⁺. However, prolonged aggregation, leading to the formation of large aggregates and fibrils, may reduce the overall concentration of permeabilizing agents, which could account for the observed decrease in permeabilizing activity in the assay.

## Conclusion

In conclusion, we have developed an ultrasensitive, single‐molecule confocal microscopy assay combined with fast‐flow microfluidics to study the permeabilization of lipid bilayers in large unilamellar vesicles (LUVs). This approach eliminates the need for vesicle immobilization and provides unparalleled temporal resolution and sensitivity, enabling the detection of fluorescence bursts at rates close to 1000 vesicles per minute. This platform enables precise quantification of membrane‐permeabilizing activity with a detection limit as low as 135 pM, making it ideally suited for studying valuable and scarce compounds. However, the overall limit of detection depends on the intrinsic activity of the tested compound. The assay's robust performance was validated through the quantification of the concentration‐dependent activities of both simple ionophores, such as ionomycin, and complex pore‐forming agents, including antimicrobial peptides (AMPs) and protein aggregates. Importantly, the assay allowed us to differentiate between leakage caused by dye release and specific calcium transport, thanks to the use of high‐molecular‐weight dextran‐conjugated calcium‐sensitive dyes. The developed assay provides a powerful tool with high‐throughput capabilities for studying membrane‐disrupting agents at low concentrations, making it well‐suited for drug discovery efforts targeting antimicrobial resistance, neurodegenerative diseases, cancer, and channelopathies.

## Supporting Information

The authors have cited additional references within the Supporting Information.^[^ ^45^, ^46^ ^]^

## Author Contributions

K.M.B.: conceptualization, methodology, validation, formal analysis, investigation, writing – original draft, writing – review & editing. D.C.E.: methodology, validation, formal analysis, investigation, data curation, writing – review & editing, visualization. D.G.: conceptualization, methodology, software, validation, formal analysis, investigation, writing – review & editing. B.S.: investigation, formal analysis. R.F.: investigation, formal analysis. T.Z.: resources. K.P.: resources. A.L.: resources. S.L.C.: conceptualization, writing – review & editing, supervision, resources, project administration, funding acquisition. M.H.: conceptualization, software, writing – review & editing, resources, supervision, project administration, funding acquisition.

## Conflict of Interests

K.P. and A.L. were employed by Stressmarq Biosciences Inc. who gifted the α‐synuclein used to generate the data in Figure 4. The authors declare that they have no other conflict of interests.

## Supporting information



Supporting Information

## Data Availability

The data that support the findings of this study are openly available in Zenodo at http://doi.org/10.5281/zenodo.15096531, reference number 15096531 and in the Supporting Information of this article. Python scripts used for data analysis are available at https://github.com/dan‐c‐edwards/Confocal‐LUV.
