# Supplementary material for: A Single‐Molecule Liposome Assay for Membrane Permeabilization
Source: Angew Chem Int Ed Engl. 2025 Jun 5;64(26):e202503678. doi: 10.1002/anie.202503678 (PMC12184303; doi:10.1002/anie.202503678)
Supplement: Supplementary file 1 — Supporting Information [file ANIE-64-e202503678-s001.pdf]

Supporting Information

A Single-Molecule Liposome Assay for Membrane Permeabilisation

Krzysztof M. Bąk,+[a] Daniel C. Edwards,+[a][b] Dylan George,+[a][b] Bhanu Singh,[a][b] Ryan Ferguson,[a][b] Tianxiao Zhao, [a][b] Kristin Piché,[c] Ariel Louwrier,[c] Scott L. Cockroft,*[a] Mathew H. Horrocks*[a][b]

[a] Dr K. M. Bąk, Dr D. C. Edwards, Dr D. George, Dr B. Singh, R. Ferguson, Prof. S. L. Cockroft, Prof. M. H. Horrocks
EaStCHEM School of Chemistry, University of Edinburgh, Joseph Black Building, David Brewster Rd, EH9 3FJ, UK.

[b] Dr D. C. Edwards, Dr D. George, Dr B. Singh, R. Ferguson, T. Zhao, Prof. M. H. Horrocks
IRR Chemistry Hub, Institute for Regeneration and Repair, University of Edinburgh, Edinburgh, EH16 4UU, UK

[c] K. Piché, Dr A. Louwrier
Stressmarq Biosciences Inc., Suite 117 -1537 Hillside Ave, Victoria V8T 2C1, British Columbia, Canada.

+These authors contributed equally to this work.

Contents

S1. Materials and Methods 2
S1.1. Preparation of vesicles 2
S1.2. NMR analysis of vesicles 2
S1.3. DLS analysis of vesicles 3
S1.4. Calculation of vesicle concentration 4
S1.5. Preparation of microfluidic device 4
S1.6. Single-molecule fluorescence confocal microscopy 4
S1.7. Preparation of α-synuclein aggregates 5
S1.8. Characterisation of α-synuclein aggregates 6
S2. Confocal measurements 8
S2.1. General procedure for permeabilisation measurements 8
S2.2. Normalisation of signals 9
S2.3. Vesicle stability studies 10
S2.4. Vesicle studies with various dyes 11
S2.5. Studies with ionomycin 13
S2.6. Studies with alamethicin 15
S2.7. Studies with melittin 16
S2.8. Studies with α-synuclein 17
S2.9. Liposome dissolution with Triton X-100 18
S3. References 18

S1. Materials and Methods

NMR spectra were recorded on a Bruker AVA 600 MHz spectrometer. Chemical shifts are reported as δ values in ppm. Dynamic light scattering (DLS) was recorded on an Anton Paar Litesizer DLS 500 using plastic cuvettes of 1 cm path length. Vesicles were prepared as described below using Avanti mini extruder apparatus, equipped with polycarbonate membranes with 200 nm pores. Vesicle purification was carried out using GE Healthcare PD-10 desalting columns prepacked with Sephadex G 25 medium. All buffers and stocks solutions prepared using biological grade water (Fisher Bioreagents). Ionomycin, melittin, POPC (Avanti), and HEPES were purchased from Sigma Aldrich. Alamethicin was purchased from Stratech Scientific. These chemicals were used without further purification.

S1.1. Preparation of vesicles

A thin film of lipid was prepared by evaporating a deacidifed (by passing through basic alumina) chloroform solution of POPC under reduced pressure on a rotavap with high rotation to provide aliquots of 6 μmol. Films were furthered dried for 6 hours under high vacuum and stored at -20°C until use. Lipid films were warmed to room temperature and mixed with prepared dye-containing buffer (dye 5 µM, 20 mM HEPES, pH 7.0) and hydrated by sonication for 30s and vortexing for 1 hour. The lipid suspensions were then subjected 10 freeze-thaw cycles using liquid nitrogen and a water bath (40 °C). Vesicles were then diluted and extruded 29 times through a polycarbonate membrane (pore size 200 nm). Extra-vesicular components were removed by size exclusion chromatography on a PD-10 sephadex G-25 column washed with prepared buffer (20 mM HEPES, pH 7.0) to yield solution of vesicles with lipid concentrations in the range of 2.5-3 mM.[1] Dye-filled vesicles are prepared fresh every day for analysis.

S1.2. NMR analysis of vesicles

Lipid concentrations were determined by ^{1}H NMR analysis as previously reported.[2] To a clean NMR tube 430 μL CD_3OD, and 175 μL of vesicle stock solution, 100 μL $CDCl_3$, and 20 μL 5 mM TMSP in D_2O were added and gently mixed. The 1H NMR spectrum was measured with presaturation to suppress the water signal (5s relaxation delay, 64 scans, 25°C). To determine lipid concentration, the TMSP reference peak (~0 ppm) integral was compared to the integral of the lipid methyl group (~0.88 ppm). Lipid concentration was calculated using **Equation S1**, where C_{St} is the concentration of TMSP (5 mM), V_{St} is the volume of TMSP (20 μL), V_{Lp} is the volume of the vesicle solution (175 μL), I_{St} is the value of the integral of TMSP, I_{Lp} is the value of the integral of lipid methyl group (~ 0.88 ppm).

$$\text{(Equation S1)} \qquad \textbf{Concentration of Lipid} = \frac{3C_{St}V_{St}I_{Lp}}{2V_{Lp}I_{St}}$$

S1.3. DLS analysis of vesicles

The size and distribution of vesicles was verified by Dynamic Light Scattering (DLS) measurements on an Anton Paar Litesizer 200 at 20 °C, using disposable cuvettes. Standard parameters were used for the vesicles and samples were diluted (1:20) in a buffer (20 mM HEPES, pH 7.0) with corresponding viscosity of 0.91 mPa·s and refractive index of 1.33. The average intensity DLS data from 3 different batches of LUVs are plotted in **Figure S1** and hydrodynamic radius values fall in the range of 75–85 nm (diameter: 150–170 nm).

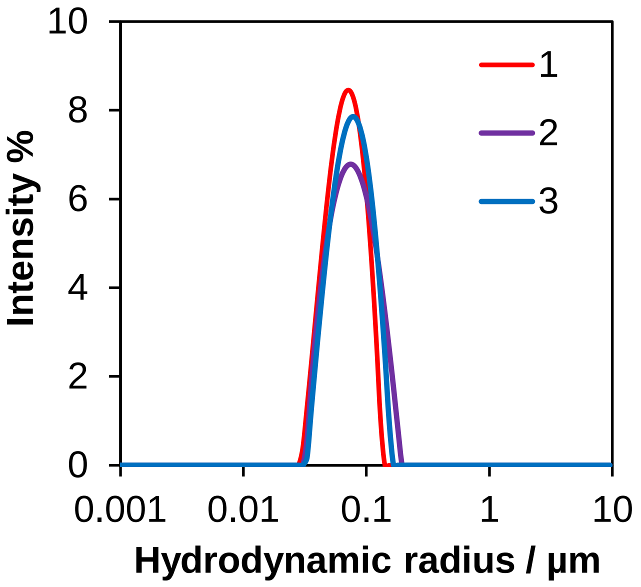

Figure S1. Hydrodynamic radius distribution of prepared vesicles from three separate batches as determined by DLS analysis.

The size and concentration of vesicles was further analysed by light scatter measurements on a Zetaview Particle Matrix at 20 °C, using disposable cuvettes. Standard parameters were used for the vesicles and samples were diluted (1:10,000) in a buffer (20 mM HEPES, pH 7.0). The particle size, concentration, and distribution of vesicles were determined at 488 nm as in **Figure S2** with a hydrodynamic diameter in the range of 150–180 nm.

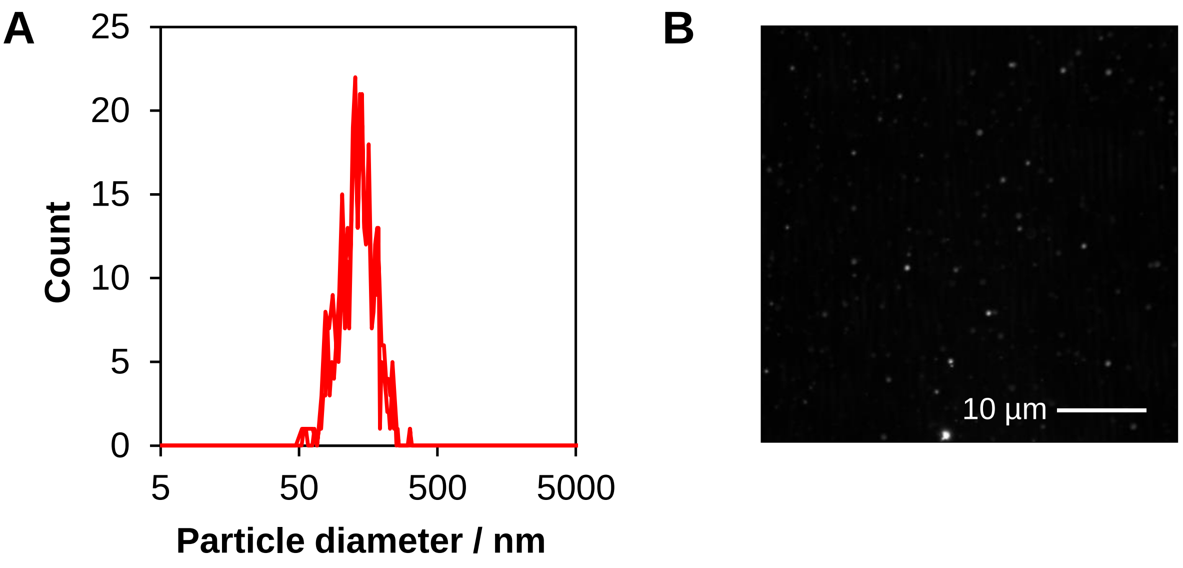

Figure S2. A. Particle diameter distribution of prepared vesicle solution. **B**. Zetaview imaging of vesicles at 488 nm scattering.

S1.4. Calculation of vesicle concentration

- Mean diameter = 180 nm = 1800 Å
- The surface area of a sphere = πd^2 , therefore the surface area of these vesicles = 1.02×10^7 Å^2
- The surface area of POPC was calculated to be 64.7 ± 1.3 Å^2 (Simulating POPC and POPC/POPG Bilayers: Conserved Packing and Altered Surface Reactivity)
- This gives the number of POPC's per vesicle as: $2\times1.02 \times 10^7/64.7$ = 3.15×10^5
- Lipid concentration of 5 µM corresponds to (5×10^{-6} M) / (3.15×10^5) = 1.58×10^{-11} M = 15.8 pM = 9.56×10^{12} L^{-1}

S1.5. Preparation of microfluidic device

The microfluidic device design has been described previously,[3] and consists of a single channel (width = 100 µm, height = 25 µm, length = 1 cm). Microfluidic devices were fabricated using standard soft-lithography techniques into polydimethylsiloxane (PDMS; Dow Corning) with SU-8 photoresist on silicon masters, as described previously.[4] The channels were oxygen plasma-bonded to glass coverslips (VWR, thickness = 1) to create sealed devices

S1.6. Single-molecule fluorescence confocal microscopy

The apparatus used for single molecule coincidence detection was similar to that previously described and shown in **Figure S3**.[5,6] A Gaussian beam at 488 nm (LBX-405-100-CSB-OE, Oxxius) was directed through the back port of an inverted microscope (Nikon Eclipse TE2000-U), where it was reflected by a dichroic mirror

(DI03-R405/488/561/635, Semrock) through an oil immersion objective (Nikon CFI Plan Apochromat VC 100x Oil, NA 1.4, W.D 0.13 mm) and focused 10 µm into the microfluidic device. Careful manipulation of the focal spot was used to find the position of vesicles flowing through the microfluidic device. Fluorescence was collected by the same objective and imaged onto a 50 µm pinhole (Thorlabs). The fluorescence was filtered by optical filters (long-pass: BLP01-488R-25 and band-pass: FF01-525/30-25, Semrock) before being focused onto an avalanche photodiode, APD (SPCM-14, Perkin Elmer, and Waltham, MA). Output from the APD was connected to a USB data acquisition card (USB-CTR04, Measurement Computing), which counted the signals and combined them into time-bins of 100 µs, the expected residence time of the vesicles in the confocal volume.

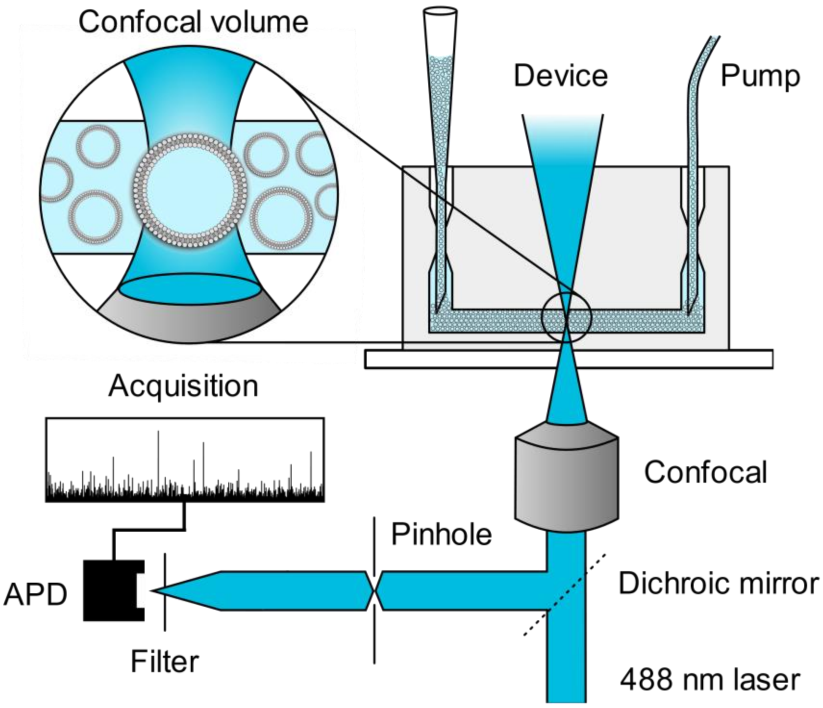

Figure S3. Schematic of the experimental setup, consisting of an inverted confocal fluorescence microscope interfaced with a flow cell. The microfluidic device was fabricated using standard soft lithographic methods.

S1.7. Preparation of α-synuclein aggregates

Aggregates formed as previously described.[6] The protein was diluted in PBS (pH 7.4) to a total protein concentration of 70 µM. The buffer was freshly prepared before each experiment and passed through a 0.02 µm syringe filter (Anotop, Whatman) to remove insoluble contaminants. The aggregation mixture was kept in DNA LoBind microcentrifuge tubes (Eppendorf) and left shaking (200 rpm) at 37 °C in an incubator for the duration of the experiment. Aliquots were taken at a series of time points over the incubation period and were immediately analysed with confocal measurements.

S1.8. Characterisation of α-synuclein aggregates

Surface preparation for SAVE imaging

Coverslips were treated with argon plasma for 45 minutes to eliminate autofluorescent organic contaminants. Frame-seal slide chambers (Biorad, SLF-0601) were attached to the glass surface, and 50 µl of poly-L-lysine (PLL, Sigma-Aldrich, P4707-50ML) was added and incubated for 30 minutes. After incubation, excess PLL was removed by washing the chamber three times with 0.02-µm–filtered PBS. A protein solution (500 nM in PBS) was applied to the surface and incubated for 15 minutes, then replaced with 5 µM ThT solution in PBS immediately before imaging.

TIRF microscopy imaging

Single-molecule imaging was carried out using a custom-built TIRF microscope, restricting excitation of fluorophores within the sample to 200 nm from the sample-coverslip interface. Collimated laser light at a wavelength of 405 nm (Cobolt MLD 405–250 Diode Laser System, Cobalt, Sweden) was aligned and directed parallel to the optical axis at the edge of a 1.49 NA TIRF Objective (CFI Apochromat TIRF 60XC Oil, Nikon, Japan), mounted on an inverted Nikon TI2 microscope (Nikon, Japan). A perfect-focus system corrected the imaging process for any stage-drift. Fluorescence was collected by the same objective and separated from the TIR beam by a dichroic mirror Di01-R405/488/561/635 (Semrock, Rochester, NY, USA). Collected light was then passed through appropriate filters (BLP01-488R-25 (Semrock, NY, USA). The emission beam was passed through a 2.5x beam expander and focussed onto an EMCCD camera for image collection (Delta Evolve 512, Photometrics, Tucson, AZ, USA) operating in frame transfer mode. Pixel size was 103 nm. Images were recorded with an exposure time of 50 ms with 405 nm excitation (~100 W cm−1). The microscope was automated using the open-source microscopy platform Micromanager (NIH, Bethesda).

The data were analyzed using custom-written code in Python 3.8 (code available at 10.5281/zenodo.7546532). The images were first averaged over the 50 frames, and the background subtracted using threshold_local in skimage.filters. Spots corresponding to ThT-bound aggregates were selected by applying a threshold equal to the mean + 5 x S.D. of the intensity in each image. The total number of aggregates were counted in each image and the data combined to give an overall count normalised to the imaging area.

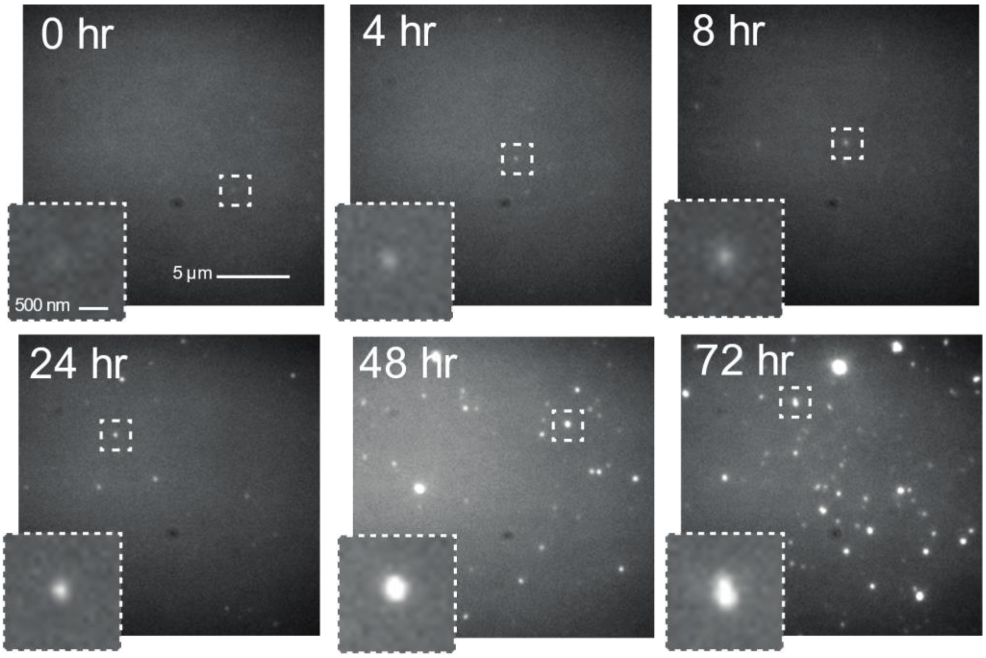

Figure S4. Processed SAVE image of α-synuclein aggregates taken at different incubation times across the range of 0-72 hrs.

S2. Confocal measurements

S2.1. General procedure for permeabilisation measurements

Dye-filled vesicles are diluted to a stock solution of 0.005 mM lipid with a buffer (20 mM HEPES, pH 7.0). A solution of calcium chloride is prepared by dissolving $CaCl_2 \cdot 3H_2O$ in biological grade water to a concentration of 22 mM. Stock solutions of permeabilising agents are prepared according to supplier instructions, diluted prior to analysis, and added to a suitable concentration range of 0.0001–10 mol% with respect to the lipid concentration. For the determination of I_{min}, 10 μL of 22 mM $CaCl_2$ solution is added to 100 μL solution of dye-filled vesicles (0.005 mM) and mixed gently. The sample is then transferred in a gel-loading tip and mounted in a device. The microfluidic channel is initially washed with this solution at a flow rate of 1000 μL/hr for two minutes. The flow rate is then reduced to 96 μL/hr for two minutes and the confocal measurements are acquired at 10,000 Hz sampling rate using a 488 nm laser at 5 mW power for five minutes.

For the determination of I_{max}, 10 μL of 22 mM $CaCl_2$ solution is added to 100 μL solution of dye-filled vesicles (0.005 mM), followed by ionomycin (**Figure S5**, final concentration 1 mol% to lipid). Such solution is then gently mixed and incubated at room temperature for 5 minutes. The sample is then transferred in a gel-loading tip and mounted in a device. The microfluidic channel is initially washed with this solution at a flow rate of 1000 μL/hr for two minutes. The flow rate is then reduced to 96 μL/hr for two minutes and the confocal measurements are acquired at 10,000 Hz sampling rate using a 488 nm laser at 5 mW power for five minutes.

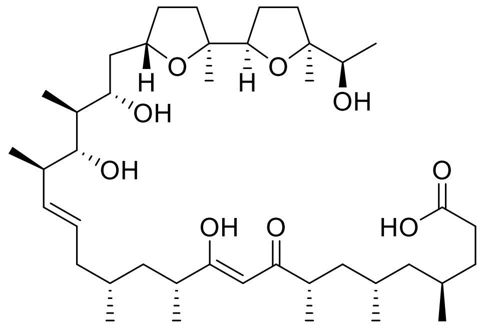

Figure S5. Chemical structure of calcium transporting agent ionomycin.

For the determination of permabilising agent activity, dye-filled vesicles (0.005 mM), $CaCl_2$ (2 mM) and the permeabilising agent (0.1–10 mol% relative to lipid) are combined and mixed by vortexing. The mixture is allowed to incubate at room temperature for a predefined amount of time (typically 10 minutes for alamethicin, melittin, and α-synuclein). The sample is then transferred in a gel-loading tip and mounted in a device. The microfluidic channel is initially washed with this solution at a flow rate of 1000 μL/hr for two minutes. The flow rate is then reduced to 96 μL/hr for two minutes and the confocal measurements are acquired at 10,000 Hz sampling rate using a 488 nm laser at 5 mW power for five minutes.

S2.2. Normalisation of signals

To minimise variation between batches of dye-filled vesicles the number of events from measurements are normalised using the f_{min} and f_{max} measurements. Values of f are calculated by analysing raw data point files collected from confocal measurements where individual photon bins are assessed based on a predefined threshold criterion. Hence, f represents the number of bins within the five-minute analysis time which have an intensity greater than the set threshold. Unless otherwise stated the threshold used for analysis if 50 photon s^{-1}. The f_{min} value corresponds to the number of events in a blank sample containing Ca^{2+} (2 mM) and dye-loaded vesicles (0.005 mM, lipid concentration). The f_{max} value indicates the number of events corresponding to fully sensitised dye signal, which is determined by the Ca^{2+} permeabilisation with ionomycin (1 mol% to lipid). The number of events from the analysed sample (f_{sample}), f_{min}, and f_{max} produce comparable normalised value of activity ($f_{normalised}$) with **Equation S2**.

Equation S2

$$f_{normalised} = \frac{f_{sample} - f_{min}}{f_{max} - f_{min}}$$

S2.3. Vesicle stability studies

For all measurements the vesicles were prepared fresh each day to mitigate effects of their degradation. Measurements of vesicle size over time indicate an increase in hydrodynamic radius and polydispersity over the course of 5 days as a result of deformation and aggregation of vesicles, **Figure S6A**. Furthermore, studies with the same batch of dye-filled vesicles show a reduction in f_{max} signal intensity (**Figure S6B**) which indicates leakage of the dye out of the lower stability vesicles and hence a reduced resolution for detection of permeabilising agents.

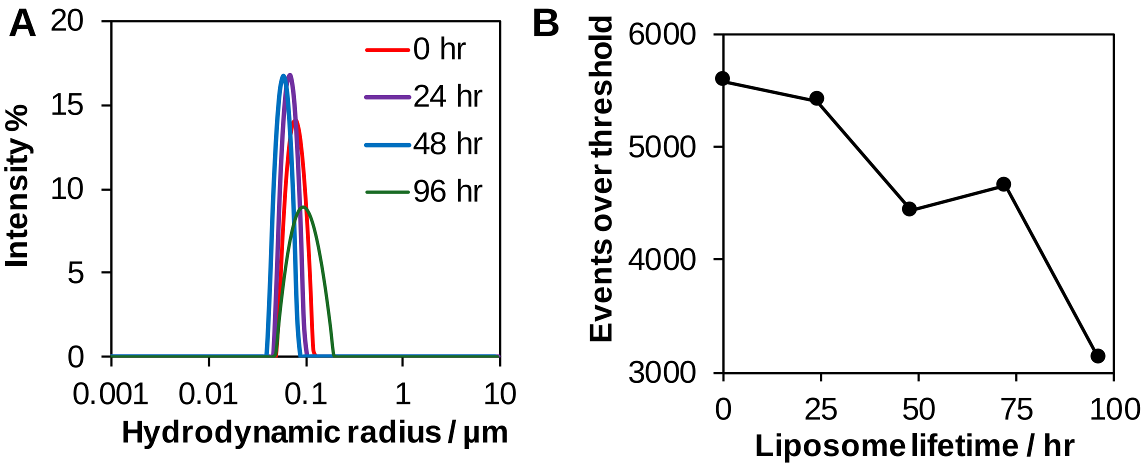

Figure S6. Degradation of vesicles over time. **A**. DLS analysis of vesicle batch immediately after preparation up after up to 5 days. **B**. Number of events detected above the threshold following the addition of ionomycin (1 mol%). The number of detected events gradually decreases to around half of the original at a lifetime of five days.

S2.4. Vesicle studies with various dyes

Two dyes were used during these studies with their structures presented in **Figure S7**. Fluo-8 is a fluorescent probe that shows spectral response upon binding of calcium and bare structural similarity to that of Cal-520-dextran. However, the addition of the dextran conjugation provides reduction in leakage and compartmentalisation, especially in the case of larger aperture channel-forming peptides and proteins.

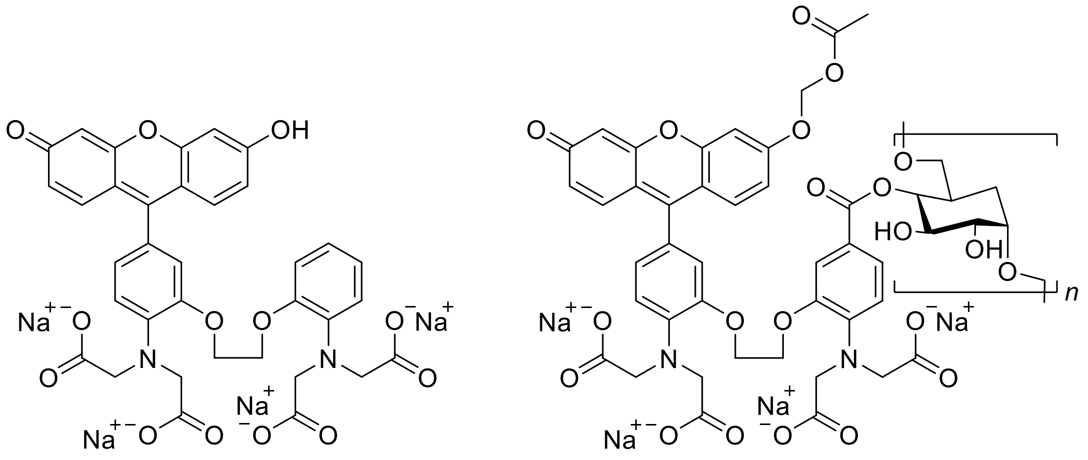

Fluo-8 Cal-520-dextran (11k)

Figure S7. Chemical structure of Fluo-8 and Cal-520-dextran dyes. Where *n* is in a range of 50-60 dextran units.

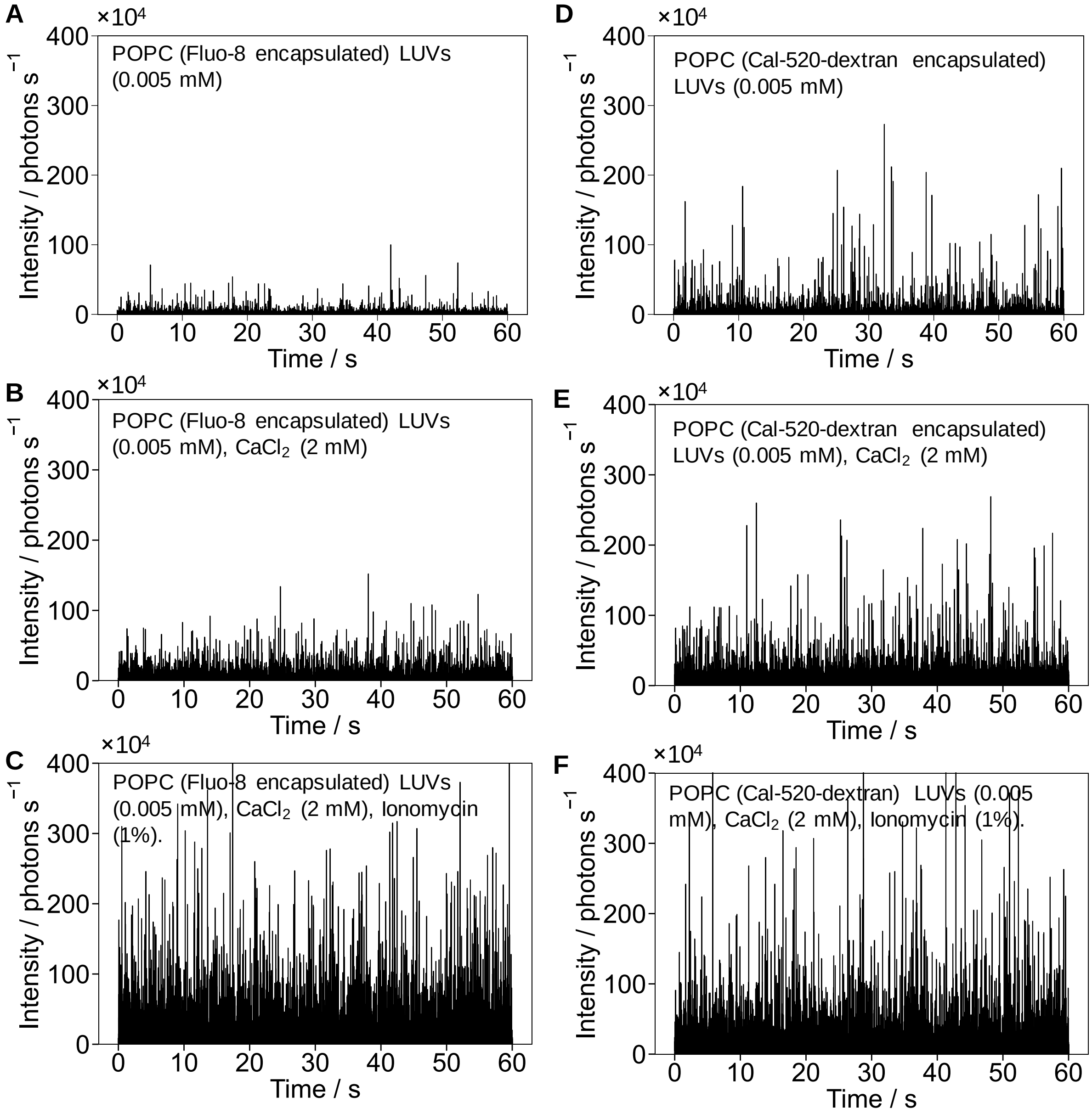

Figure S8. A. Example of data collected over one minute for Fluo-8 dye-filled LUVs in presence of $CaCl_2$. **B**. Example of data collected over one minute for Fluo-8 dye-filled LUVs in presence of $CaCl_2$. **C**. Example of data collected over one minute for Fluo-8 dye-filled LUVs in presence of $CaCl_2$ and ionomycin. **D**. Example of data collected over one minute for Cal-520-dextran dye-filled LUVs. **E**. Example of data collected over one minute for Cal-520-dextran dye-filled LUVs in presence of $CaCl_2$. **F**. Example of data collected over one minute for Cal-520-dextran dye-filled LUVs in presence of $CaCl_2$ and ionomycin.

S2.5. Studies with ionomycin

To estimate the limit of detection for ionomycin, the standard deviation of $f_{normalised}$ for a blank sample was calculated by repeating blank measurements three times in three independent batches of vesicles. For each batch standard deviation of sample was calculated, and then averaged across the three batches (**Table S1**).

Table S1 Values of $f_{normalised}$ and their standard deviations recorded for three independent blank samples.

	**Blank 1**	**Blank 2**	**Blank 3**
**Repeat 1**	-0.02708	0.02596	0.00892
**Repeat 2**	-0.00903	0.02642	0.00538
**Repeat 3**	-0.02888	0.05194	0.00769
**Average**	-0.02166	0.03477	0.00733
**Standard Deviation**	0.02822		

The limit of blank (LoB) is the highest apparent number of counts expected to be found when replicates of a sample containing no analyte are detected, and is given by the expression:[7]

Limit of blank LOB = mean blank + 1.645 × (SD of blank) = 0.006813 + 1.645 × 0.02822 = 0.053238

Table S2 Values of $f_{normalised}$ and their standard deviations recorded for three independent batches at low concentrations of iononmycin.

	**0.001 %mol**	**0.0025 %mol**	**0.005 %mol**
**Repeat 1**	0.03791	0.04513	0.16787
**Repeat 2**	0.05104	0.08921	0.14160
**Repeat 3**	0.05353	0.07468	0.17807
**Standard Deviation**	0.00840	**0.02246**	0.01881

The limit of detection (LoD) was determined by utilising both the measured LoB and test replicates of a sample known to contain a low concentration of analyte, and is defined as:

Limit of detection LOD = LOB + 1.645 × (SD of low concentration sample) = 0.090

The highest standard deviation among the three batches (0.02246) was used as an estimate of the standard deviation a low concentration sample.

LOD of 0.090 corresponds to **~0.0027 %mol of ionomycin** (based on Hill analysis).

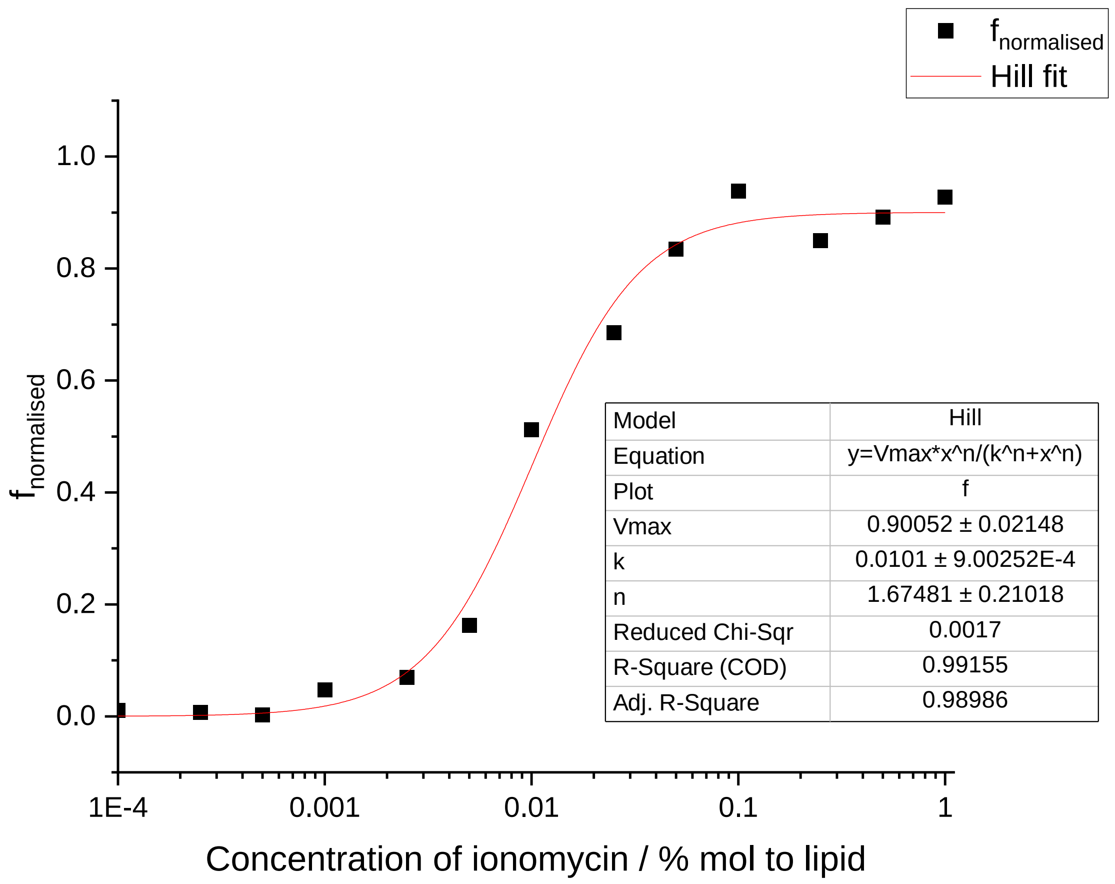

Figure S9. Hill analysis of ionomycin activity. EC50 = 0.0101 ± 0.0009.

S2.6. Studies with alamethicin

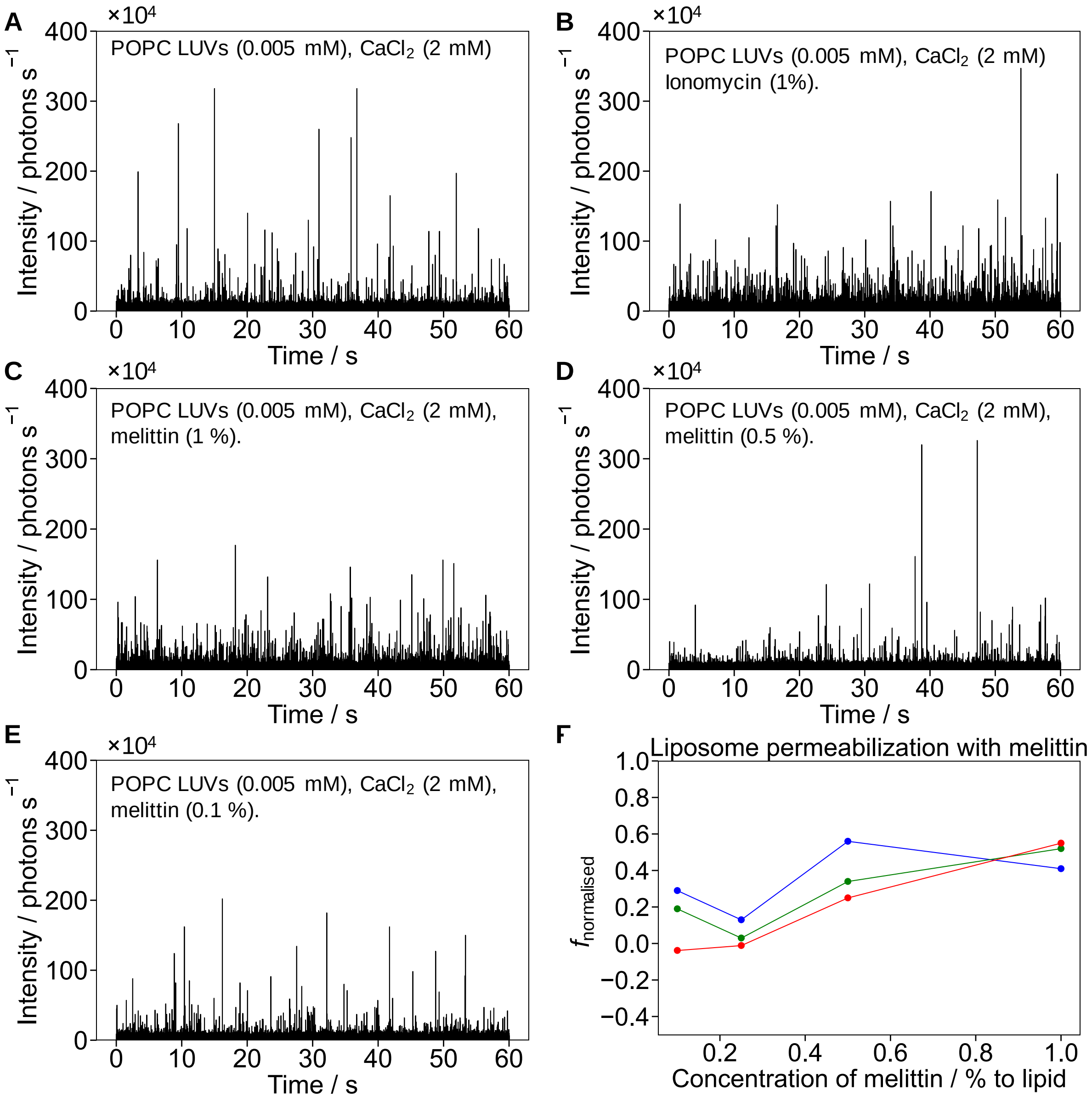

Figure S10. Permeabilisation studies with alamethicin. Analysis conducted using POPC vesicles filled with Cal-520-dextran dye. **A**. Example of data collected over one minute for Cal-520-dextran dye-filled LUVs in presence of $CaCl_2$ (to calculate f_{min}). **B**. Example of data collected over one minute for Cal-520-dextran dye-filled LUVs in the presence of $CaCl_2$ and ionomycin (to calculate f_{max}). **C-E**. Example data collected over one minute for Cal-520-dextran dye-filled LUVs in the presence of $CaCl_2$ and varying concentrations of alamethicin. **F**. Plot of vesicle permabilisation with alamethicin measured in three independent batches at various concentrations with signals normalised using **Equation S2**. Concentration reported as %mol relative to the amount of lipid.

S2.7. Studies with melittin

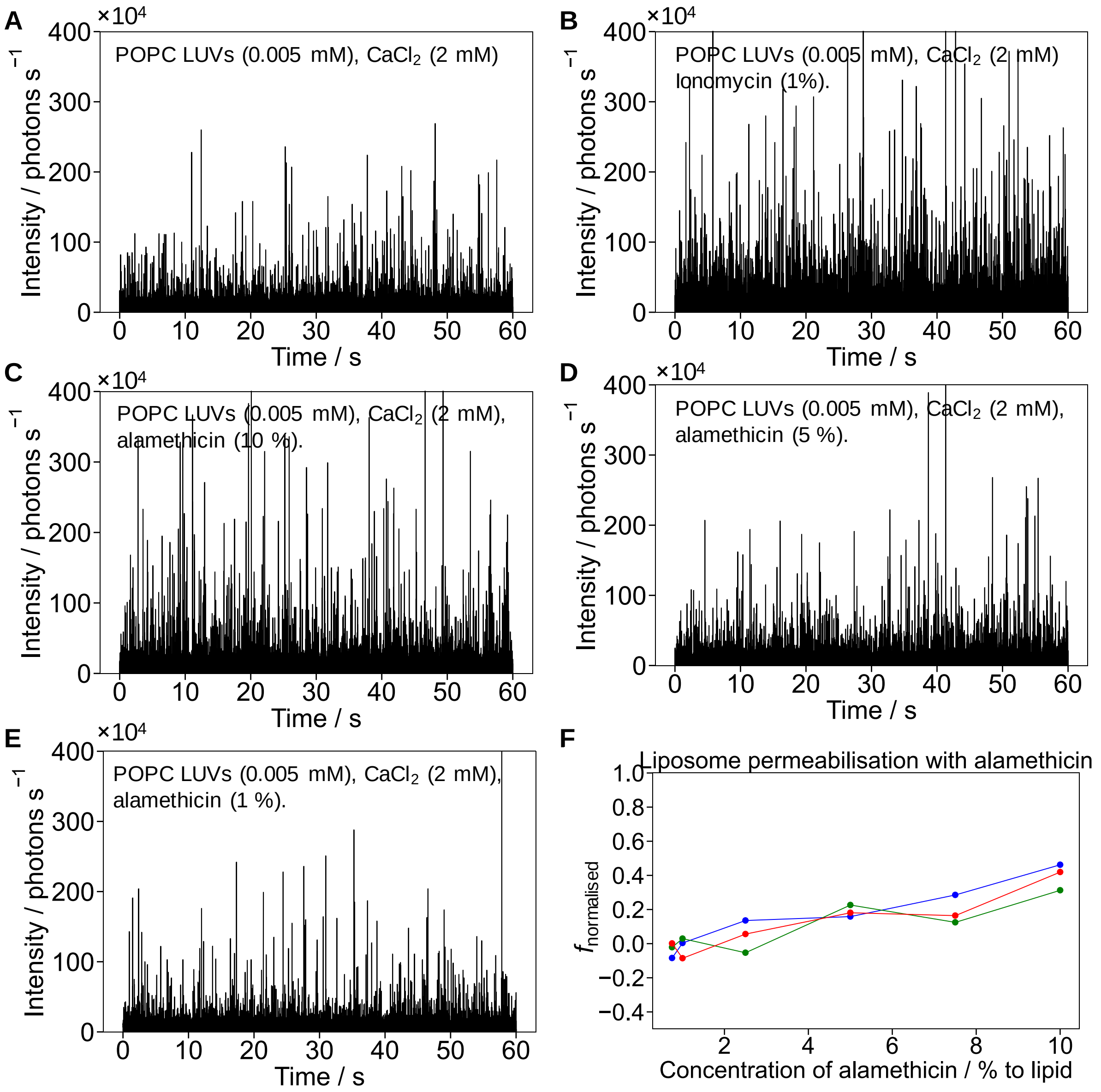

Figure S11. Permeabilisation studies with melittin. Analysis conducted using POPC vesicles filled with Cal-520-dextran dye. **A**. Example of data collected over one minute for Cal-520-dextran dye-filled LUVs in the presence of $CaCl_2$ (to calculate f_{min}). **B**. Example of data collected over one minute for Cal-520-dextran dye-filled LUVs in the presence of $CaCl_2$ and ionomycin (to calculate f_{max}). **C-E**. Example of data collected over one minute for Cal-520-dextran dye-filled LUVs in the presence of $CaCl_2$ and varying concentrations of melittin. **F**. Plot of vesicle permabilisation with alamethicin measured in three independent batches at various concentrations with signals normalised using **Equation S2**. Concentration reported as %mol relative to the amount of lipid.

S2.8. Studies with α-synuclein

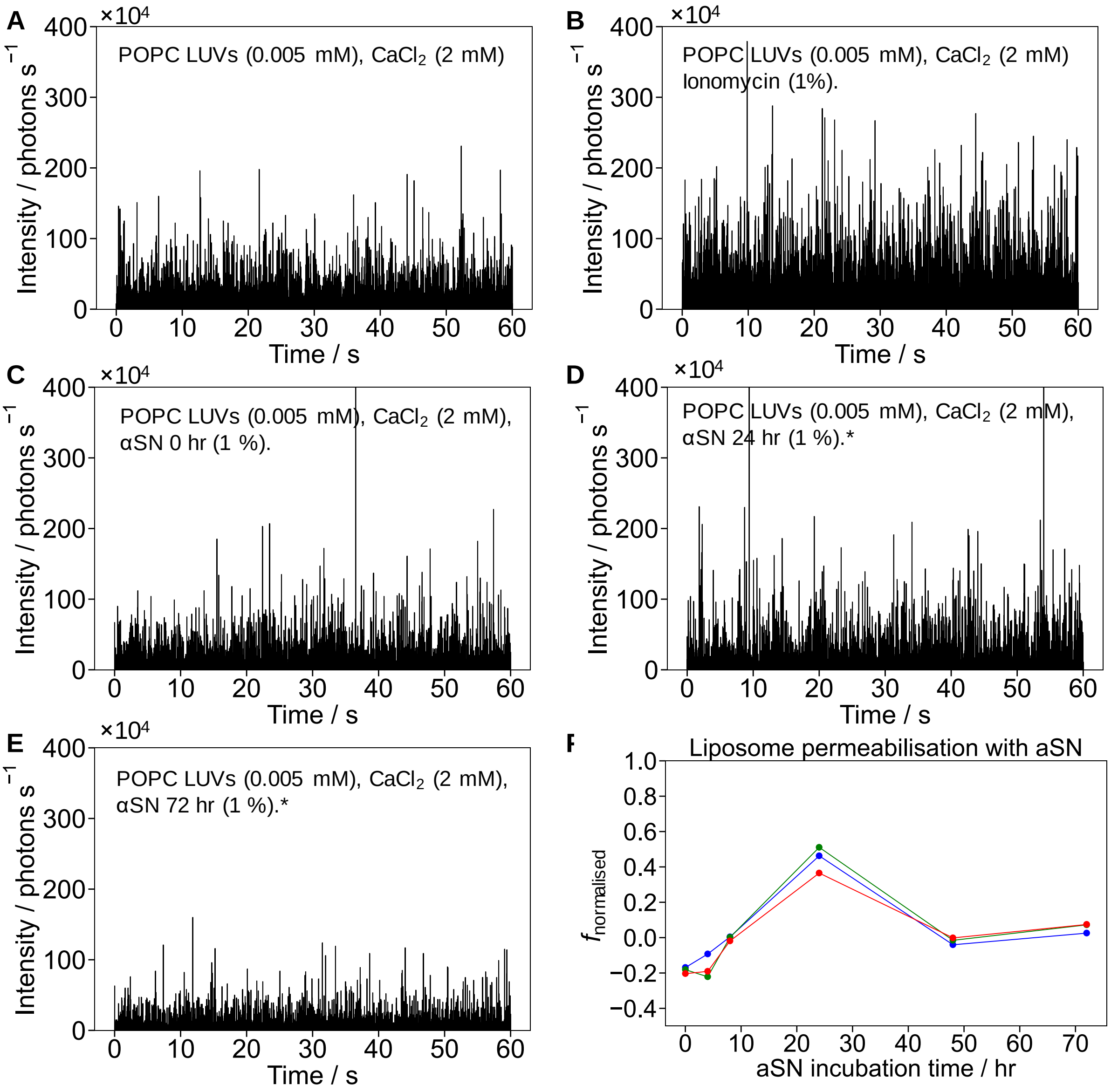

Figure S12. Permeabilisation studies with aggregates of α-synuclein. Analysis conducted using POPC vesicles filled with Fluo-8 dye. **A**. Example of data collected over one minute for Fluo-8 dye-filled LUVs in the presence of $CaCl_2$ (to calculate f_{min}). **B**. Example of data collected over one minute for Fluo-8 dye-filled LUVs in the presence of $CaCl_2$ and ionomycin (to calculate f_{max}). **C-E**. Example of data collected over one minute for Fluo-8 dye-filled LUVs in the presence of $CaCl_2$ and α-synuclein after varied incubation times. **F**. Plot of vesicle permabilisation with α-synuclein, measured in triplicate at various incubation times, with signals normalised using **Equation S2**.

S2.9. Liposome dissolution with Triton X-100

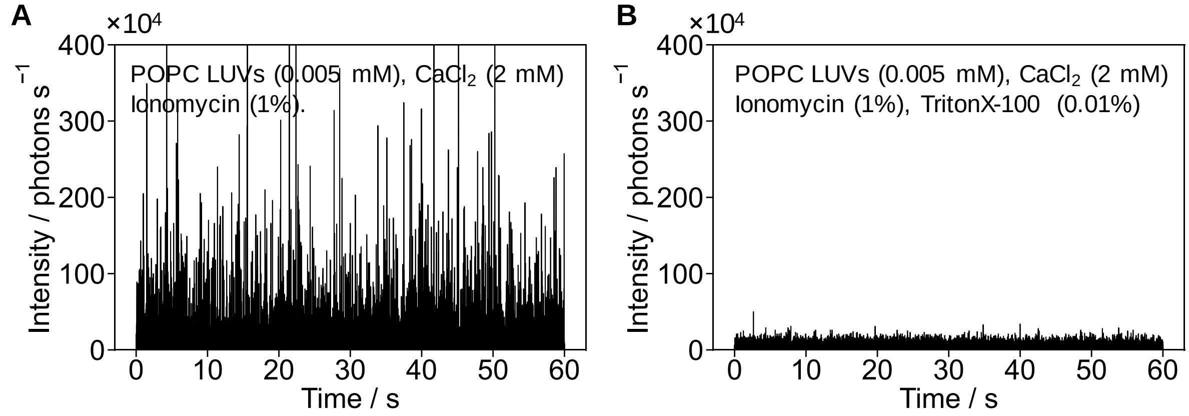

Figure S13. Demonstration of change in signal intensity following dissolution of dye-filled LUVs with Triton X-100. **A**. Example of data collected over one minute for Fluo-8 dye-filled LUVs in the presence of $CaCl_2$ and Ionomycin (1%) **B**. Example of data collected over one minute for Fluo-8 dye-filled LUVs in the presence of $CaCl_2$ and ionomycin (1%) prepared in a buffer containing Triton X-100 (0.01 %).

S3. References

(1) Chvojka, M.; Singh, A.; Cataldo, A.; Torres-Huerta, A.; Konopka, M.; Šindelář, V.; Valkenier, H. The Lucigenin Assay: Measuring Anion Transport in Lipid Vesicles. *Anal. Sens.* **2024**, *4*, e202300044. https://doi.org/10.1002/anse.202300044.

(2) Hein, R.; B. Uzundal, C.; Hennig, A. Simple and Rapid Quantification of Phospholipids for Supramolecular Membrane Transport Assays. *Org. Biomol. Chem.* **2016**, *14*, 2182–2185. https://doi.org/10.1039/C5OB02480C.

(3) Horrocks, M. H.; Tosatto, L.; Dear, A. J.; Garcia, G. A.; Iljina, M.; Cremades, N.; Dalla Serra, M.; Knowles, T. P. J.; Dobson, C. M.; Klenerman, D. Fast Flow Microfluidics and Single-Molecule Fluorescence for the Rapid Characterization of α-Synuclein Oligomers. *Anal. Chem.* **2015**, *87*, 8818–8826. https://doi.org/10.1021/acs.analchem.5b01811.

(4) Horrocks, M. H.; Li, H.; Shim, J.; Ranasinghe, R. T.; Clarke, R. W.; Huck, W. T. S.; Abell, C.; Klenerman, D. Single Molecule Fluorescence under Conditions of Fast Flow. *Anal. Chem.* **2012**, *84*, 179–185. https://doi.org/10.1021/ac202313d.

(5) Orte, A.; Clarke, R.; Balasubramanian, S.; Klenerman, D. Determination of the Fraction and Stoichiometry of Femtomolar Levels of Biomolecular Complexes in an Excess of Monomer Using Single-Molecule, Two-Color Coincidence Detection. *Anal. Chem.* **2006**, *78*, 7707–7715. https://doi.org/10.1021/ac061122y.

(6) Chappard, A.; Leighton, C.; Saleeb, R. S.; Jeacock, K.; Ball, S. R.; Morris, K.; Kantelberg, O.; Lee, J.-E.; Zacco, E.; Pastore, A.; Sunde, M.; Clarke, D. J.; Downey, P.; Kunath, T.; Horrocks, M. H. Single-Molecule Two-Color Coincidence Detection of Unlabeled Alpha-Synuclein Aggregates. *Angew. Chem. Int. Ed.* **2023**, *62*, e202216771. https://doi.org/10.1002/anie.202216771.

(7) Armbruster, D. A.; Pry, T. Limit of Blank, Limit of Detection and Limit of Quantitation. *Clin. Biochem. Rev.* **2008**, *29*, S49−S52.
